# Intercalated Intermetallic Compounds AlTi_3_ and Fe_2_Ti in Microrods and Microtubes Obtained by Invariant Reaction of Mechanically Milled System Al_43_Ti_36_Fe_21_

**DOI:** 10.3390/ma12233806

**Published:** 2019-11-20

**Authors:** Lucía G. Díaz Barriga Arceo, Leonardo González Reyes, Jesús Noé Rivera Olvera, Abraham Medina Ovando, Vicente Garibay Febles

**Affiliations:** 1 Depto. Ingeniería en Metalurgia y Materiales UPALM ESIQIE IPN, Apartado Postal 118-018, Del. GAM, Ciudad de México C.P. 07051, Mexico; 2 Departamento de Ciencias Básicas, Universidad Autónoma Metropolitana-Azcapotzalco, Av. Sn. Pablo No. 180, Ciudad de México 02200, Mexico; lgr@azc.uam.mx; 3 Tecnológico Nacional de México, Campus Ixtapaluca. TESI, Km. 7 de la carretera Ixtapaluca-Coatepec s/n, Ixtapaluca, Estado de México C.P. 56580, Mexico; 4 ESIME Azcapotzalco, Instituto Politécnico Nacional. Av. de las Granjas 682, Col. Sta. Catarina, Azcapotzalco 02250, Ciudad de México, Mexico; amedinao@ipn.mx; 5 Programa de Ingeniería Molecular, I.M.P. Lázaro Cárdenas 152, Ciudad de México C.P. 07730, Mexico; vgaribay@imp.mx

**Keywords:** mechanical alloying, decomposition, invariant reaction, rods, tubes, nanostructures

## Abstract

This paper reports the production of intermetallic microrods and microtubes from the decomposition of an intermetallic compound in an AlTiFe system. The intermetallic compound was obtained by mechanosynthesis of elemental powders of Al, Ti and Fe over 300 h at 400 rpm, sintering from compacted powder particles at 300 MPa per minute and at 900 °C for 3600 s in an argon atmosphere. The milled and sintered samples were analyzed by X-ray diffraction (XRD), scanning electron microscopy (SEM) and high-resolution transmission electron microscopy (HRTEM). The intermetallic AlTi_3_ and Fe_3_Al phases were obtained during the milling process. After sintering, a decomposition of these intermetallic phases was found—Al_3_Ti_0.75_Fe_0.25_, Al_3_Ti, FeTi, AlTi_3_, Ti_9_Al_23_, Fe_2_Ti, Al_86_Fe_14_ and Al_0.4_Fe_0.6_. As a result of the decomposition, we observed the formation of hexagonal rods with intercalated phases of AlTi_3_ and Fe_2_Ti.

## 1. Introduction

Micro and nanometric sizes of nanotubes, rods and fibers with large lengths show exceptional physical properties due to their geometry. Their mechanical, thermal, optical and electrical properties are often exceptional; therefore, these materials are used for the design of miniature electronic and electromechanical devices and for use in electronic and bioelectronic sensors, solar cells, light-emitting diodes or as reinforcers in composite materials [1,2,3,4,5,6,7,8,9,10]. These materials are commonly prepared using physical and chemical methods. These rods are mainly composed of ceramic and transition metal oxides, which have been extensively analyzed [4]. Jiawen et al. [5] synthesized lanthanum hydroxide with rod-like morphology by hydration processing via the hydration of its bulk oxide in normal water solution at boiling temperature. The rods had a width of 2–3 μm and a length of 5–8 μm. Li et al. [6] reported the production of GaN with rod morphology, metalorganic vapor phase epitaxy, with a width of 1 μm and a length of 5.9 μm. Gromyko et al. [7] prepared ZnO with different morphologies, including rods, disks and nanosheets, via electrodeposition. They produced rod-like structures with an average diameter of 200 nm and a length of up to 1 μm. Zhao et al. [8] prepared a one-dimensional (1D) ZnO nanorod array via electrochemical deposition (ECD) processed in a two-electrode cell. A single-crystal structure of ZnO nanorods with the growth direction along the [002] direction was observed. The literature reviewed above only reported the production of ceramics and nitrides with a rod array. Reports of the synthesis of metal rods and aluminum alloys or intermetallic rods are lacking. The production of two-phase intermetallic rods caused by some invariant decomposition reaction that promotes unidirectional solidification was reported [4,9]. Unidirectional solidification can be induced by heat treatments around an invariant reaction point. In these thermal treatments, some different crystalline structures can be formed—intercalated compounds, sheets of different intercalated phases, columnar structures that may or may not be facetted and globular structures of the core shell type. The formation of columnar structures is limited; however, the production of this kind of structure requires a simple and low-cost method that allows the study of the microstructure and properties of many systems, including intermetallic systems [5,6,7,8,9,10,11,12,13,14,15,16]. Intermetallic systems show interesting properties compared with those of alloys because their properties, depending on their composition, are an intermediate between an alloy and a ceramic. In particular, Ti aluminides, such as AlTi, AlTi_3_ and Al_3_Ti, are used in high-temperature-resistant coating applications as they have a high melting point, high mechanical strength, low density and excellent resistance to chemical attack at high temperatures. The main limitation in their application is their limited ductility and their fragility at room temperature but the addition of transition elements, such as Fe, N, Cr or V, improves their ductility and increases their resistance to corrosion [11]. The AlTiFe system is an interesting intermetallic due to its mechanical and chemical resistance properties but it is a ternary alloy that requires very high preparation temperatures (between 1800 and more than 2000 K) [17,18,19]. However, the AlTiFe mixture is associated with intermediate binary phases with triple points that may be of interest for directional solidification studies [19].

The purpose of the present work was to demonstrate that it is possible to obtain intermetallic rods via mechanosynthesis and sintering, in particular for the Al_43_Ti_36_Fe_21_ system. Mechanical alloying (MA) is a solid-state powder technique involving repeated welding, fracturing and rewelding of powder particles in a high-energy ball mill. It is an effective synthesis method for preparation of a wide range of materials including for preparing intermetallic phases like the AlTi–M system, where M is another metal, such as Cu, Fe, Si, Mn or others. The benefits of this technique (MA) have been acknowledged since the 1990s. After this milling process, the composite powders were compacted and sintered under atmospheric conditions. In this last step, we explain how the growth of nanometric particles used as nucleation sites for rod formation is induced when the system is near or at the invariant reaction point [16].

## 2. Materials and Methods

We used Al, Ti and Fe powders with purities greater than 99% as raw materials. The powders were mixed with a nominal composition of 43 wt %, 36 wt % and 21 wt %, respectively. The raw materials were processed in a low-energy ball mill for 300 h with 400 rpm rotation. The powders and media milling of spherical-shaped zirconia (φ = 20 mm) were loaded and sealed in stainless steel inside a glove box, first, with a vacuum and then, with an argon atmosphere. Powder handling was conducted under atmospheric conditions and the ball-to-powder ratio (BPR) was 10:1. To avoid agglomeration in the powders, methanol was used as the process control agent (PCA). Then, milled powders were compacted in a uniaxial press with a pressure of 262.6 MPa, forming half-inch-diameter green compacts. The samples were encapsulated in quartz tubes under an argon atmosphere and heat-treated at 900 °C for one hour in a Linderg Blide M tubular furnace (Thermo Scientific, Waltham, MA, USA). Then, the samples were tempered in water with ice [20,21,22]. The raw materials and processed samples were analyzed in a D8 Focus diffractometer (Bruker, Karlsruhe, Germany), with a graphite monochromator and CuKα radiation (λ = 0.1542 nm) at a speed of 2°/min. The phases present in all the samples were identified using the International Centre for Diffraction Data Powder Diffraction Files ICDD PDF-2 crystallographic database (2003). We analyzed the chemical and rod morphology using energy-dispersive X-ray spectroscopy (EDS) under a high-resolution scanning electron microscope (SEM; model JSM 6701F, JEOL, Tokyo, Japan). The peaks were indexed and the crystalline structure of the rods was observed with a high-resolution electronic microscope (80–300 kV; FEI-Titan Microscope, Hillsboro, OR, USA) using the spherical aberration coefficient correction technique (Cs). For this purpose, the samples were prepared in an SEM assisted by a focused beam of gallium ions SEM/FIB JEM-4501 (JEOL).

## 3. Results and Discussion

### 3.1. X-Ray Diffraction Microstructural Characterization

**Figure 1:**
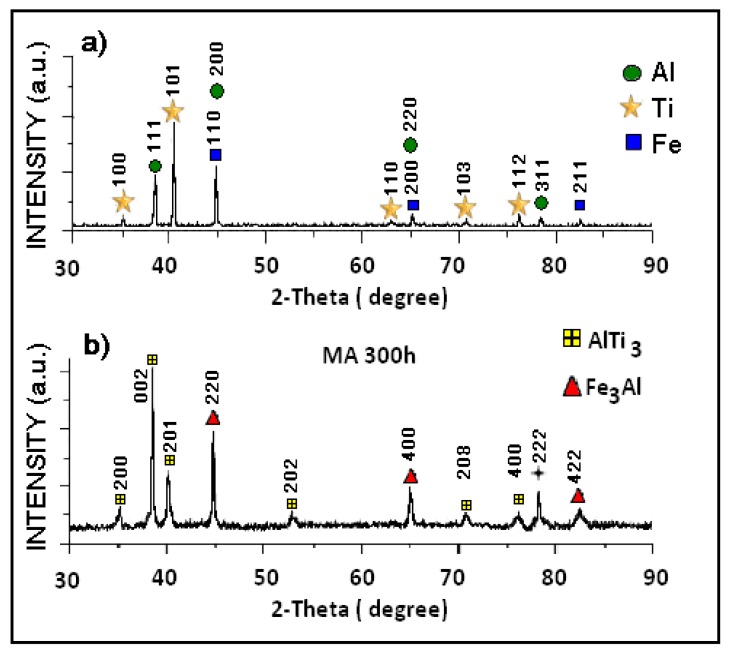
X-ray diffraction (XRD) patterns of (**a**) the mixture of Al, Ti and Fe powders and (**b**) after milled for 300 h.

**Figure 2:**
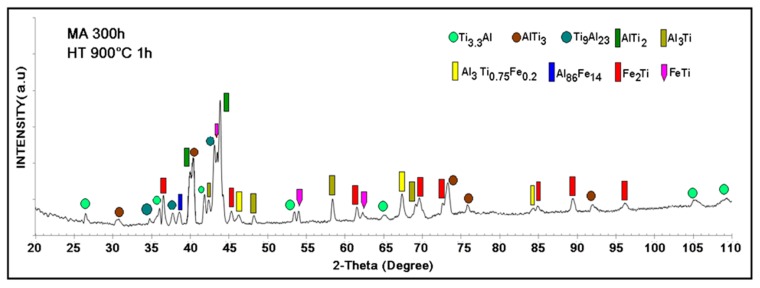
XRD results of the Al_43_Ti_36_Fe_21_ system obtained by mechanical alloying for 300 h and heat-treated at 900 °C for one hour.

Figure 1 and Figure 2 show the X-ray diffraction (XRD) patterns of the mixture of elemental powders (Figure 1a), powders milled for 300 h (Figure 1b) and powders milled and sintered at 900 °C for one hour (Figure 2). Figure 1a depicts the diffraction pattern of the initial powder mixture and the characteristic peaks of Al, Ti and Fe. The crystalline structure indexed in these powders is shown in Table 1. Figure 1b displays the phases formed by mechanical alloying after 300 h; the following phases were identified—AlTi_3_ and Fe_3_Al. These phases had already been reported using this preparation method [23]. Fe contamination was 0.5% by weight and was determined by atomic absorption. The crystal size of the sample milled for 300 h was calculated according to Scherrer’s equation: τ = Kλ/βcosθ, where λ, θ, β and K are the X-ray wavelength, the Bragg diffraction angle, the line broadening at half the maximum intensity and the constant related to crystallite shape, respectively [23,24]. We calculated that the average crystal sizes for AlTi_3_ were about 63.2 nm and 32.3 nm for Fe_3_Al.

In the sample milled for 300 h, the compositions in wt % of the formed phases were calculated considering the relative intensities of the peaks of the diffraction pattern in Figure 1b using the reference intensity ratio (RIR) method. We qualitatively determined that the intermetallic compound AlTi_3_ with a hexagonal structure was formed and had 68.11 wt % and Fe_3_Al with a cubic structure centered in the body reached 31.89 wt %. We observed the broadening of the peaks after 300 h of milling, which is related to the reduction in the particle size due to the intense deformation of the powders due to the repeated phenomena of deformation, fracture and cold welding in the mechanosynthesis process. Table 1 and Table 2 show all the phases identified by the XRD results of the samples either unmilled or milled at 300 h and the respective weight percentage composition calculated using the RIR relative intensities method. Figure 2 depicts the diffraction pattern of the sample milled for 300 h and compacted and then heat-treated at 900 °C for one hour. The spectrum shows all the phases obtained after the heat treatment. The Fe_3_Al phase formed during the milling process, as shown in Figure 1, does not appear after sintering; its decomposition generated the transformation to Fe_2_Ti and FeTi phases and the AlTi_3_ phase, which is the major phase obtained in the mechanosynthesis, was partially decomposed to form other intermetallic compounds Ti_9_Al_23_, AlTi_2_ AlTi_3.3_ and Al_3_Ti. In the decomposition, the intermetallic compounds obtained in the milling were mixed and formed Al_86_Fe_14_ and Al_3_Ti_0.75_Fe_0.2_.

The additional detail shown in the diffractogram in Figure 2 is the overlapping of some peaks associated with several phases, which was expected because it is a decomposition reaction and because the phases were expected to be intercalated, as occurs, for example, in systems with eutectic reactions [19]. Table 2 outlines the different phases in the sample milled for 300 h and thermally treated at 900 °C for 1 h. Every phase in Table 2 has a crystalline structure and a lattice parameter associated with the XRD results.

### 3.2. Scanning Electron Microscopy

**Figure 3:**
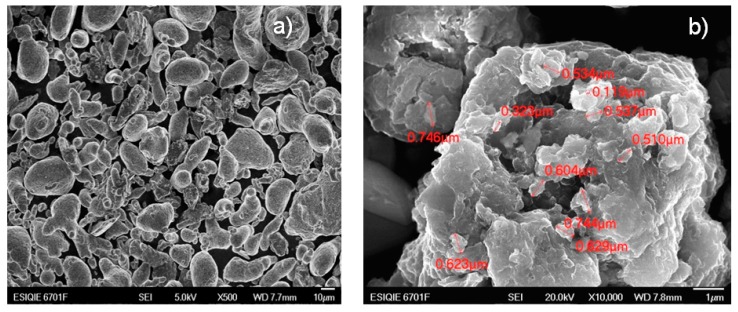
(**a**) The mixture of raw materials of Al, Ti and Fe powders and (**b**) Al, Ti and Fe powders after 300 h of milling.

The micrograph in Figure 3a shows the initial mixture of the elemental metal powders of Al, Ti and Fe. Different morphologies are observed that can be easily identified by observation—lamellar, rounded and flat surface particles; in Al, particles with irregular and angular edges with a rough surface belong to Ti; and lamellar particles with rounded edges and irregular surface are Fe. The diameters of the powders are larger than 10 microns.

**Figure 4:**
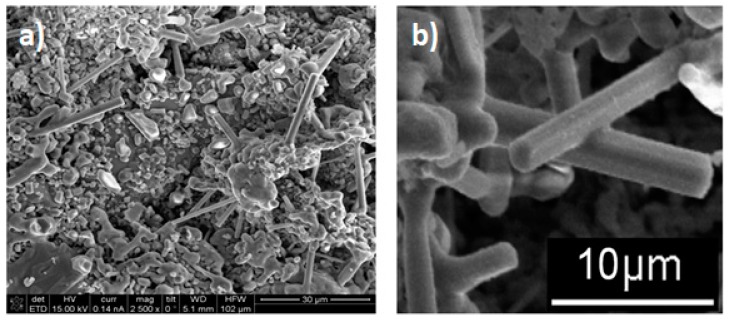
(**a**) Sample sintered at 900 °C for one hour and (**b**) formation of intermetallic microrod of the AlTiFe system.

Figure 3b shows a micrograph of the sample milled at 300 h, where lamellar structures stacked with average diameters of about 874 nm can be observed, with the nanocrystals’ diameters ranging between 20 and 60 nm. The changes in the powders milled at 300 h and sintering at 900 °C in these samples were noticeable and are presented in Figure 4a,b.

**Figure 5:**
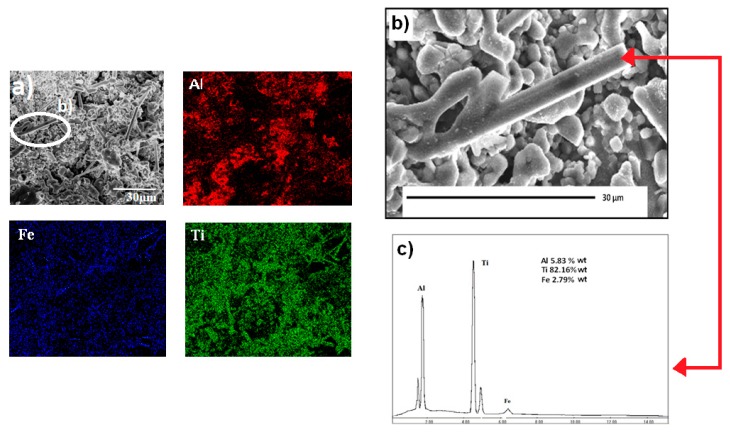
(**a**) Scanning electron microscopy (SEM) elemental mapping of Al_43_Ti_36_Fe_21_ system powder sample, milled for 300 h and heat-treated at 900 °C. (**b**) Zoom of selected area of a rod and (**c**) energy dispersive x-ray spectroscopy (EDS) analysis with SEM on the selected spot of an intermetallic rod.

**Figure 6:**
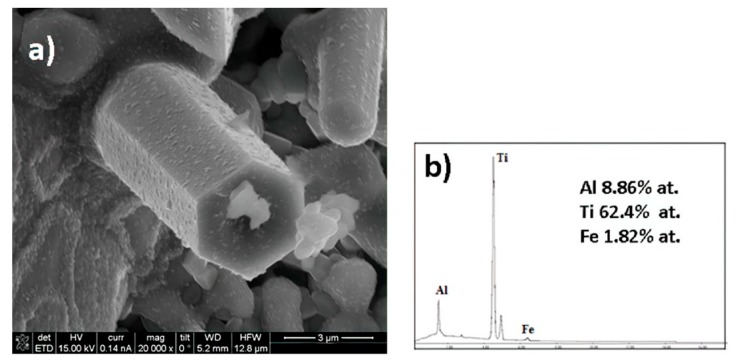
(**a**) SEM micrograph and (**b**) EDS analysis of an intermetallic rod formed from mechanosynthesis and heat treatment of the Al_50_Ti_30_Fe_20_ system.

**Figure 7:**
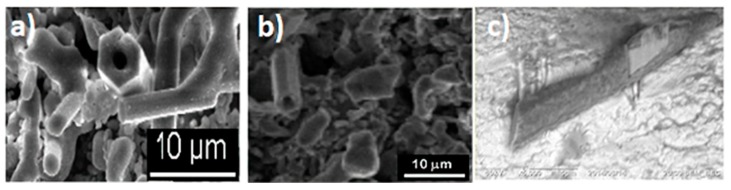
(**a**) Fe_2_Ti microtubes with a width of 2 µm and intermetallic microtubes with a width of 5 µm in the AlTi_3_ system, (**b**) AlTi_3_ microtubes with a width of 3 µm and (**c**) longitudinal cut of the rod that has two phases.

The micrographs in Figure 4a,b depict the formation of rod structures with hexagonal morphology. These rods are longer than 10 microns and the diameters ranged between 500 nm and 2 µm. A careful observation of Figure 4b shows that some of these rods have ramifications, which are usually associated with the presence of twinning defects. Figure 5a shows these same elongated crystals with a chemical mapping performed under a scanning microscope, where the distribution of Al, Ti and Fe from the rods, are observed. The mapping on the rods, especially those located in the upper-right part of the micrograph, show a homogeneous distribution of Ti and Fe and some homogeneous dispersion of Al but with lower intensities. Figure 5b shows the chemical analysis of a spot on a rod from the micrograph circle zoom in Figure 5a. The presence of Al, Ti and Fe can be observed in the EDS spectrum, suggesting that the three elements are present in the rod (see Figure 5c). According to the X-ray diffraction pattern, the phase containing these three elements is Al_3_Ti_0.75_Fe_0.2_ but this phase is cubic (Table 2) and does not correspond to the hexagonal shape of the rods, as shown in Figure 6 and Figure 7a, which suggest that the rods are of hexagonal crystalline symmetry. This phenomenon is explained by phase intercalation as expected and observed in the XRD pattern in the peaks exhibiting phase overlapping. In the case of hexagonal phase overlapping, we inversed the following crystalline systems possibilities in the rods—AlTi_3_/Fe_2_Ti and AlTi_3.3_/Fe_2_Ti. Figure 6 and Figure 7a also show the hexagonal phase in the rods, which is only observable at a specific diameter. If the rods show widths less than 800 nm, the hexagon is not observed; conversely, hexagonal structures are clearly observed if the rod is between 900 nm and 3.5 µm wide. Lengths are variable and range between 3 µm and longer than 10 µm.

EDS spot showing the location of the energy dispersive x-ray spectroscopy (EDS) analysis of an intermetallic rod formed from mechanical alloying and heat treatment of the Al50Ti30Fe20 system.

Evidence of the differences in the concentrations of the AlTi_3_ and Fe_2_Ti phases in the rods can be observed in the formation of tubular structures in the system, as shown in Figure 7a,b. Continuous rods with a smooth surface and hexagonal tubes were observed, without observing their interior due to the differences in concentration of the phases mentioned above. However, we suspect that a small core of the Fe_2_Ti system exists around which the hexagonal shell of AlTi_3_ grows. This description is consistent with the X-ray diffraction results in Figure 2 that were analyzed in Table 2.

The hexagonal core of these rods formed by the decomposition of the Fe_3_Ti phase to form the FeTi and Fe_2_Ti phases. According to a study of phase transitions in FeTi alloys by Chien and Li [21], the FeTi phase can be amorphous and under certain conditions of compaction and heating, it is transformed into an FeTi cubic phase or an Fe_2_Ti hexagonal phase, which is very compact. In this study, heating and compacting occurred in the presence of the Al_3_Ti phase. This phase may control the nucleation of the Fe_2_Ti phase and its diffusion. Figure 7 depicts the formation of branches in non-hexagonal rods and the presence of twins, which could indicate the transformations of the intermetallic compounds with Fe and Ti. We may also have produced rods with an Al_3_Ti core and an Fe_2_Ti shell. The type of rod depends on the concentration of each elemental powder at the time of growth. Some rods were not perfectly hexagonally shaped, which could explain why not all rods have the same morphology. This is due to the fact that the compositions of the rods depend on which side of the invariant reaction point the different concentrations of these formations are found.

### 3.3. High-Resolution Transmission Electron Microscopy (HRTEM)

**Figure 8:**
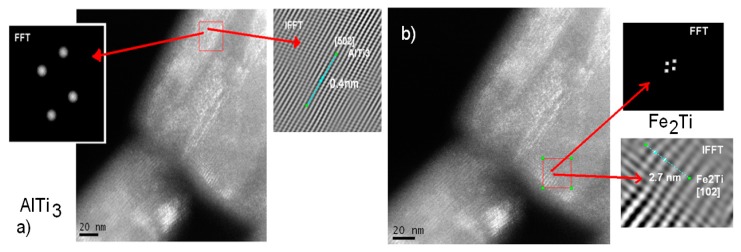
Darkfield analysis of the phases in the rods (**a**) on one side and (**b**) in the center of the rods. We observed that the center is formed mainly by the Fe_2_Ti phase and the side by the AlTi_3_ phase. The contrast of the photo indicates intercalations of these phases, especially on the side of the rod.

The SEM/FIB (Scanning Electron Microscope/Focused Ion Beam) micrograph in Figure 7c shows the result of a lateral cut on one of the thinnest rods. We analyzed it with backscattering electrons (from SEM/FIB). As shown in the image (Figure 7c), the center of the rod is brighter, which indicates that at least two types of phases exist in the material with different densities. The slice taken from this rod was analyzed using high-resolution microscopy in darkfield mode, to facilitate visualization of the crystalline lattice within the rod. The following micrographs show the phases found in the center and the side of the rod (see Figure 8). The analysis was conducted with the help of the Digital Micrograph program in fast Fourier transform (FFT) mode, where a crystalline plane of the material was selected in the diffraction space and inverse fast Fourier transform (IFFT), where the plane can be observed in real space. In the plane, the interplanar spacings were measured in one direction, the crystallographic database was consulted and the phase was assigned. Magnification of the inset figure on one side of the rod (see Figure 8a) as simulated, reveals that the d-spacing is 0.400 nm, which corresponds to AlTi_3_ phase and [502]. The electron diffraction pattern simulated from the crystalline region and [102] associated with this d-spacing is indexed as the Fe_2_Ti phase with a hexagonal structure and lattice parameters a = 7.85 nm and c = 4.29 nm. Hence, from Figure 8a,b HRTEM, we observed after indexing that the rods in their outermost region are formed mainly with the AlTi_3_ phase, which is hexagonal and the center of the Fe_2_Ti phase rods. According to the X-ray diffraction database, the AlTi_3_ phase has a molecular weight of 170.68 g/mol and Fe_2_Ti has a molecular weight of 159.59 g/mol. With the HRTEM results and the image in Figure 7c shows that the brightest areas are rich in AlTi_3_ and those with the lowest brightness are rich in Fe_2_Ti (as observed in Figure 7c). These results are in accordance with the observations of the XRD results and SEM images of the backscattering electrons. The contrast in the photo indicated the intercalation of phases outside the core of the rod, which is expected for decomposition reactions.

**Figure 9:**
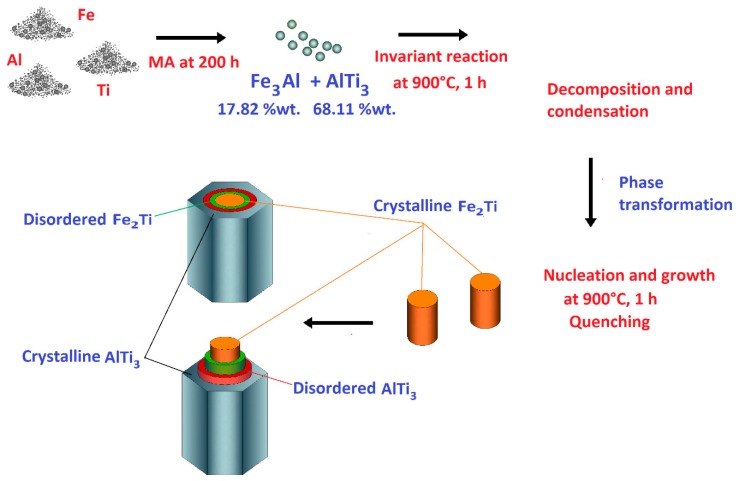
Schematic illustration of the evolutions of phase and rod formation during mechanical alloying and sintering at 900 °C.

In addition, we tried to obtain similar results reported above by milling the system Al_43_Ti_36_Fe_21_ at 200 and 400 h with the same sintering conditions. However, no rod structure formations were observed. This indicates that the formation of AlTi_3_ and Fe_3_Al phases with a nanometric particle size with 68.11 wt % and 31.89 wt %, respectively, compacted at 262.6 MPa and sintered at 900 °C is an invariant reaction point and that their decomposition results in unidirectional solidification. At 200 and 400 h of grinding, the phase percentages are different. The results for 200 and 400 h are not reported here to focus on the production of rods at 300 h and to shorten this paper. Figure 9 shows the evolution of phases and formation of rods during mechanical alloying, sintering at 900 °C and quenching of the powder particles of Al, Ti and Fe. The proposed mechanism of formation of these rods is as follows—(1) The milling process at 200 h causes a reduction in crystal size to the nanometric level and introduces crystalline defects. The direct transformation to AlTi_3_ and Fe_3_Ti occurs during mechanical alloying, as reported in the literature [25]. (2) A decomposition of the previous phases and condensation of the smaller particles (obtained by the milling process) during sintering at 900 °C in an invariant reaction occurs with a composition of 17.82 wt % and 68.11 wt % for Fe_3_Ti and AlTi_3_, respectively. (3) Nucleation of crystalline Fe_2_Ti phase, formed mainly in the center of the rods (Figure 7a and Figure 9), can be observed as two rods with a 1 µm diameter, which corresponds to Fe_2_Ti. This is the same diameter of the hole (1 µm) found in the rod according to the micrograph in Figure 7a. (4) The crystalline Fe_2_Ti phase grows due to the formation of crystalline defects, which mainly include screw dislocation and terraces, which commonly allow the columnar growth with cubic and hexagonal structures. This growth mechanism is well known in the literature [26]. An intercalated disordered phase of FeTi is proposed to exist in the rod, since, under the condition of composition in this experimental work, with sintering at 900 °C and quenching, the powder particles were transformed into an Fe_2_Ti hexagonal phase [21]. The interfaces between the ordered and disordered FeAl (system similar to this work) in a theoretical modeling were proposed by Šesták et al. [27]. A recombination of elements (Ti, Al and Fe) and the formation of secondary phases can occur at invariant reaction points. Then a nucleation of these secondary phases from non-equilibrium are diffused until reaching a large volume in the solid solution on the matrix, where the phase grows until reaching a concentration of equilibrium [28]. (5) The formation of AlTi_3_ around the ordered–disordered Fe_2_Ti phase is probably caused by a magnetic repulsion between both phases, which did not allow the production of homogeneous AlTiFe phase in the rod. The transformation during the sintering process can be explained by the Gibbs free energies of the formation of Fe_2_Ti and AlTi_3_, which are −234.540 and −332.090 kJ/mol, respectively. Both are negative, which indicates that these species can be produced at 900 °C. We propose that intercalated phases from the center to the edge of the rod observed in different SEM micrographs follow the sequence, as illustrated in Figure 9—ordered Fe_2_Ti → disordered Fe_2_Ti → disordered AlTi_3_ → ordered AlTi_3_.

The literature reports that, depending on the thermal treatment and speed of cooling, the events expected for the rod formations are the epitaxies and eutectic reactions that efficiently form lamellar structures, rods and globular and acicular structures in solids. The growth of columnar structures is enabled by a mechanism called crystalline internal growth (intergrowth of crystals); one of its mechanisms is that networks are interspersed at grain boundaries to form high-energy defects, one of which is known as twinning. Twinning has a very high order that is not evident until temperature is applied and crystal growth occurs in that area [18,19,29]. In two-phase systems exhibiting eutectic growth, the columnar solid formed is usually composed of the two phases at different concentrations. This makes the rods thus obtained commercially attractive because, depending on their components, the properties of the rod in different directions will differ. For example, in the rods obtained in this work, electrical conductivity or magnetic properties may behave differently in the axial and longitudinal directions, which may be of interest in electronic devices.

## 4. Conclusions

This work demonstrated that the mechanosynthesis process of the Al_43_Ti_36_Fe_21_ system allowed the formation of nanometric particles that were 874 nm on average, in a compound with 68.11 wt % AlTi_3_ and at 31.89 wt % Fe_3_Al. This compound, after being sintered, showed an invariant decomposition reaction in several phases, among which the AlTi_3_ phase and Fe_2_Ti are prominent due to the formation of hexagonal core-shell columnar structures. We found that the core of the rods were the Fe_2_Ti phase and the shell mainly consisted of AlTi_3_. The AlTi_3_ phase governs the preferential growth of the rods and moderates the intermetallic phase transformations with Fe and Ti, which promotes as the initiators of growth. These structures are 20 µm long and have an average diameter of 500 nm.

## Figures and Tables

**Table 1:** Crystallographic data of the initial powders.

Metal	PDF Card Number	Phase	Lattice Parameters
Al	65–2869	Cubic F	a = 4.049 Å
Ti	89–2762	Hexagonal	a = 2.951 Å, c = 4.684 Å
Fe	65–4899	Cubic I	a = 2.867 Å

**Table 2:** Percentage by weight of phases in samples milled for 300 h and milled for 300 h and sintered at 900 °C for 1 h.

Milled for 300 h (a) Milled and Sintered at 900 °C (b)	Phase	Wt %	Structure Lattice Parameters (Å)
a,b	AlTi_3_	68.11 (a) 17.82 (b)	Hexagonal a = 5.79, c = 4.64
a	Fe_3_Al	31.89 (a)	Cubic F, a = 5.791
b	AlTi_3.3_	17.29	Hexagonal a = 11.52, c = 4.65
b	Al_23_Ti_9_	6.96	Tetragonal I, a = 3.89, c = 33.46
b	AlTi_2_	9.84	Hexagonal a = 7.85, c = 4.79
b	Al_3_Ti	10.05	Tetragonal I, a = 3.84, c = 8.59
b	Fe_2_Ti	20.02	Hexagonal a = 7.85, c = 4.29
b	FeTi	9.01	Cubic P a = 2.97
b	Al_86_Fe_14_	2.11	None reported
b	Al_3_Ti_0.75_Fe_0.2_	6.74	Cubic P a = 3.94
